# Supplementary material for: Galectin-3 did not associate with malaria-related insulin resistance in diabetic and non-diabetic respondents at a Ghanaian General Hospital
Source: PLoS One. 2025 Aug 13;20(8):e0330068. doi: 10.1371/journal.pone.0330068 (PMC12349726; doi:10.1371/journal.pone.0330068)
Supplement: Attachment — Submitted filename: REesponse to reviewers comments--plos one.docx [file pone.0330068.s002.docx]

**RESPONSE TO REVIEWERS COMMENTS**

Please, the boldened information in Arial font size 12 are the exact comments made by the esteemed Reviewers. We have presented our response in font size 12 of Times New Roman in all cases, please. whereas the reviewers comments are left aligned, our responses are in a justified format, please.

**Reviewer #1: Dear Authors,
Your manuscript addresses an important topic and contains valuable data. However, before it can be considered for publication, it needs to address several major concerns related to structure, clarity, and scientific rigor. Below are my detailed comments and questions:

1. Line Numbers: The manuscript lacks line numbers, which makes it difficult to refer to specific sections. Please include them in the revised version to facilitate the review process.**

Thanks a lot for this observation. However, as our esteemed Reviewer may be aware, manuscripts are prepared to adhere to journals requirements. Because this suggestion is not required by the journal, we are unable to respond favourably, please.

**2. Language and Clarity: The manuscript requires extensive language editing. Many sentences are overly long, ambiguous, or grammatically incorrect. We strongly recommend having a native English speaker or a professional academic editor review and edit the manuscript.**

We have carefully considered this observation by our esteemed Reviewer. Unfortunately, we find this comment to be quite vague or ambiguous because not a single example was provided to support either the perception of ambiguity or the grammatical incorrectness. This makes it difficult for us to respond appropriately to the probable expectations of our esteemed Reviewer.

Ambiguity and grammatical incorrectness will clearly impair comprehension of the write-up. Considering the presentation by our esteemed Reviewer, the perceived vagueness and grammatical incorrectness must have been quite extensive in our earlier submission. This is where we respectfully disagree with our esteemed Reviewer, since we did not find any extensive issue of vagueness and grammatical incorrectness in our earlier submission, please.

Several factors including emotions (Hamedi et al., 2020; Scrimin & Mason, 2015; Bohn-Gettler & Rapp, 2011) can affect one’s comprehension of a text. After a careful relook at our manuscript, we have come to a conclusion that when our manuscript is kindly read again by our esteemed Reviewer, in the right mood, the observation of several sentences being ambiguous or grammatically incorrect will be revised, please.

On the issue of the length of the sentences, again, a clearer suggestion than this could have been more helpful to us although we have attempted to address all such concerns, please. Several factors influence the length of a sentence in any academic writing. For example, the intended idea of the writer and the complexity of the text used are critical although it is acknowledged that, shorter sentences improve readability (Matthews & Folivi, 2023) and probable comprehension. A length of more than 35 words has even been suggested to impair readability (Matthews & Folivi, 2023). We, therefore, adopted this 35-word threshold as a guide in presenting the revised manuscript. However, as already indicated, this threshold was used as a guide since the idea we intend to communicate was also considered in our revision.

Considering our work being a comparative study, the frequent use of conjunctions should be expected which may affect the eventual length of some of the sentences. We believe that contextual use of the guide should be more appropriate in our case. We have highlighted in yellow background, the various instances where this 35-word suggestion could not be strictly adhered to in our revised manuscript for ease of reference. In all, we had 8 of such instances, which we strongly believe communicate clearly the intent of the authors, without any impairment to comprehension for the reader, in spite of the length, please. Therefore, not all the yellow background highlighted texts represent a modification in text, please.

The suggestion by our esteemed Reviewer to seek the services of a native English speaker or a professional academic editor for our manuscript is not a bad idea. However, we find it inappropriate in our case, please. This is because the extent of grammatical incorrectness and sentence ambiguity as portrayed is a bit exaggerated in our respectful view, please. Moreover, we have adequately addressed the concern of the unduly long sentences, especially, in the discussion section of the manuscript, please. With these measures in place, we respectfully, do not see the need for a language editor for our revised manuscript, please.

In as much as we do not intend to sound boastful, we kindly submit that we are Ghanaians with more than 20 years training in the English language. In Ghana, the official language of communication and teaching in all schools at all levels is the English language. As an academic and the corresponding author of this manuscript, I have more than 18 years’ experience of teaching with the English language at the University level after acquiring all my prior trainings in the English language. Whereas we may not boast of 100% error-free manuscript, we can confidently indicate that we have adequate knowledge in reading, writing and comprehension in the English language to enable us communicate with clarity, scientific findings from our research work. This is the background that gives us the confidence to indicate that we do not require the services of an external individual or group in this context. It is this same confidence which makes us consider the observation of many sentences being ambiguous, or grammatically incorrect by our esteemed Reviewer to be quite exaggerated, please.

Notwithstanding the above, we note the preference of our esteemed Reviewer for shorter sentences. However, we would like to kindly implore him/her to make room for variability in writing styles, please. This is particularly important when such perceived long sentences are grammatically correct and convey the exact idea of the writer with the needed clarity, please.

**3. The abstract should be rewritten for clarity and completeness.**

We have carefully considered this comment by our esteemed Reviewer. However, we find it to be difficult to understand in terms of the kind of clarity and completeness being requested, please. We are of the respectful view that our abstract is clear enough without the need for any modification, please. We have therefore maintained it as it was in the earlier version of the manuscript, please.

**4. Phrases like “could neither predict” are vague and need rephrasing for clarity.**

We have carefully considered this view and thoroughly searched through our manuscript for the use of the phrase “could neither predict”. We could find only one instance, where that phrase was used. The specific instance can be found in the results section of the abstract, please. Here, we disagree with our esteemed Reviewer that it is vague, please. The exact sentence is “Galectin-3 could neither predict HOMA-B nor HOMA-IR in any of the study groups.” This sentence in our respectful view is very clear without the need for any modification. The use of that phrase is appropriate because we conducted a regression analysis to find out if galectin-3 could predict HOMA-B or HOMA-IR. However, our regression analyses did not show galectin-3 to be a predictor (explanatory variable) for the observed levels of HOMA-IR or HOMA-B. This is exactly what we sought to communicate as clear as we could and we think that we have done just that, please. We therefore find it difficult to see anything ambiguous about this statement to warrant a modification, please. We believe the phrase in question is correct, unambiguous, and must be maintained in the revised manuscript, please.

**5. Please clearly state whether Galectin-3 elevation is indicative of an inflammatory state or if it reflects an independent mechanism.**

We have carefully considered this observation by our esteemed Reviewer and are of the respectful opinion that we cannot clearly make the above statement without evidence from our work. The evidence provided in our manuscript is that galectin-3 did not associate with insulin resistance in our context. In all the instances that we cited evidence of association between galectin-3 and specific disease conditions (Foko et al., 2022; Li et al., 2022; He et al., 2017; Li et al., 2016; Ohkura et al., 2014; Oakley et al., 2009) in our work, we did not come across any indication of galectin-3 being necessarily correlated with an inflammatory marker. As accuracy is important in this endevour, we have reported what is available in literature for verification. We believe that galectin-3 as a glycoprotein, may mediate its actions through interaction with other pathways which may include inflammation. However, neighter our work nor those cited in our work did report that galectin-3 elevation is indicative of an inflammatory state. We think that the manner of presentation of galectin in our write-up is a reflection of what pertains in the existing literature that we have cited, please.

**6. The statement, “Galectin-3 is a chimeric galectin found in vertebrates,” is unclear and should be rewritten for accuracy and clarity.**

Please, we have carefully considered this comment by our esteemed Reviewer and have reproduced the exact statement in the manuscript below:

“Galectin-3 is a chimeric galectin found in vertebrates [6, 7] and expressed by several tissues, including the gut, spleen, colon, kidney, and inflammatory cells such as mast cells, neutrophils and macrophages [6].”

Looking at the above sentence, we are not sure of the additional level of clarity needed again, please. We, therefore, believe respectfully that, there is nothing wrong with the sentence as far as clarity is concerned, please.

With respect to accuracy, we have kindly provided the sources (references 6&7 of the revised manuscript) for verification if in doubt. Beyond sources already cited in the manuscript, other reports (Bahreiny et al., 2024; Li et al., 2022) have been perused. In all instances, we are fully convinced that the above statement is accurate, please. Therefore, we could not do anything to it than to maintain it in the revised manuscript as expected, please.

**7. Clearly specify the type of study conducted (e.g., cross-sectional, observational) early in the introduction.**

The study type was clearly indicated in the last sentence of the introduction section in the earlier version of our manuscript and has been maintained in the revised version, please. We believe that the current location is the most appropriate, please.

**8. It would strengthen the manuscript to include data on the prevalence and burden of diabetes and malaria in your specific country or region, if available.**

We very much agree with our esteemed Reviewer with regards to this observation. However, the regional statistics provided reflect the situation in Ghana and therefore appropriate, please.

**9. The hypothesis at the end of the introduction is vague and poorly articulated. A more focused and testable hypothesis is needed, as the current ambiguity negatively affects the coherence of the entire manuscript.**

We have defined the association we sought to investigate in the revised manuscript, please. The modified version can be found in the last sentence of the introduction section on page 4 of the revised manuscript, please.

**10. The inclusion and exclusion criteria require clearer explanation. For example:
• Why were people who had a COVID-19 vaccination history and those who had received vaccinations excluded?**

**• What is the regional COVID-19 vaccination policy, and are there vaccines in use that could mimic natural infections?**

We have carefully considered the above comments by our esteemed Reviewer and have reproduced the exact section covering the inclusion and exclusion criteria below, please.

“Participants were adults of both gender, aged 20 years and above, with or without malaria, without a history of COVID-19 but vaccinated against the condition. Pregnant and nursing mothers, and individuals with hepatitis, human immunodeficiency virus (HIV), pancreatitis, bacterial infections, rheumatoid arthritis, asthma, heart failure, T1DM, diabetes insipidus, liver and kidney diseases were excluded as these conditions can influence the measured biomarkers.”

Please, from the above extract, we did not exclude those with a history of COVID-19 vaccination because all our samples had actually been vaccinated against the COVID-19 virus. We rather excluded those who had a history of COVID-19 infection because prior COVID-19 infection in itself is known to be associated with insulin resistance, several months after recovery (Obeng et al., 2024; Adatsi et al., 2024; Adatsi et al., 2023; Chen et al., 2022).

The expectation of the Ghanaian government is to have all its citizens vaccinated against the COVID-19, please. We did not come across any evidence of any COVID-19 vaccine in Ghana that mimics natural infections, please. We took that decision deliberately to avert any probable influence of COVID-19, please.

**11. It is unclear whether participants had active malaria infections at the time of enrollment or only past exposure. Please clarify.**

Please, the entire write-up including the title portray an active form of malaria and we have clearly indicated this throughout the work.

**12. You should explicitly state the rationale for excluding individuals with autoimmune diseases such as rheumatoid arthritis (RA) and type 1 diabetes mellitus (T1DM).**

Please, as indicated in our response to comment 10, RA can influence a number of the measured biomarkers in our study. With respect to T1DM, we made a decision to focus on T2DM because insulin resistance is not a risk to the development of T1DM. Thus, per the focus of our study, participants with T1DM or any condition that with the slightest potential to influence the level of any of the measured biomarkers were deliberately excluded, please.

**13. You mention calculating prevalence ratios for malaria and diabetes. Was it assumed these groups were independent? If comparisons were made between diabetics and non-diabetics with and without malaria, the difference needs to be explicitly and clearly described.**

Please, these prevalence values used were obtained from independent studies. The sources of such information have been clearly provided in the manuscript, (reference numbers 1 & 2 of the revised manuscript), please.

**14. The threshold used to define insulin resistance (HOMA-IR > 2.6) should be referenced and justified, ideally based on prior studies in a comparable population.**

Please, this had already been done in the earlier version of the manuscript (reference number 21) and has been maintained in the revised version as expected (page 7 of revised manuscript).

**15. No details were provided about whether important confounders such as age, sex, body mass index (BMI), physical activity, or socioeconomic status were adjusted for in the analysis. These are important factors that could influence the results.**

The statistical analysis section (page 9 of revised manuscript) has been modified to reflect this, please.

**16. The logistic regression results indicate that glucose and insulin were the only predictors of insulin resistance, but the outcome is expected given that HOMA-IR is calculated from these two variables. We should acknowledge this limitation.**

Although our results reflected these, they are not limitations per se because it does not happen in all cases, please. Indeed, we checked for collinearity to ensure that there is none in building the final model, please. Moreover, biological explanations exist among insulin level, insulin resistance and glucose level in any given individual as discussed in our manuscript, please. To this end, we do not the results of our regression analysis as a real limitation, please.

**17. You stated, “The serum galectin-3 level was generally higher in participants with malaria and lowest in non-diabetic participants without malaria.” This sentence is confusing and should be rewritten for clarity.**

Please, in table 2 of the revised manuscript (pages 12-14), the evidence to this statement can be found. A careful study of the values of galectin-3 across the four groups of participants give that indication, please. Considering the statement, we have a difficulty in identifying the challenge, please. Of the four groups of participants, those that had malaria showed higher levels of galectin-3. However, when the multiple comparison test was done, non-diabetic participants without malaria had the lowest level of galectin-3 compared with the other groups. This is what has been presented, please. We believe that there is adequate clarity, please.

**18. There appears to be no stratification or matching between groups for key variables such as age, sex, BMI, or physical activity. These differences may confound the observed associations.**

Please, the issue of matching of key variables does not arise because the logistic regression controlled for such confounders. In terms of stratification according to those key variables, the number of sample that will be obtained per variable in each group will not be suitable for the regression model, please.

**19. Galectin-3 is an inflammatory marker. However, no inflammatory or oxidative stress markers (e.g., CRP, IL-6, TNF-α) were included. Without these, it is difficult to contextualize galectin-3 elevation in relation to systemic inflammation or metabolic syndrome.**

This observation is well noted. However, in our case, we were specifically interested in how galectin-3 associates with insulin resistance. Since our focus was not on the relationship between galectin-3 and the indicated inflammatory markers, we believe the lack of assessment of these specific inflammatory does not take anything away from our work, please. Inflammation and oxidative stress may mediate the relationship between galectin-3 and insulin resistance. However, we did not even find our intended linear relationship between galectin-3 and insulin resistance in the current study to enable us explore that possibility as part of our discussion, please. We believe that another study beyond the current one could be designed to consider this issue of contextualization of galectin-3 in relation to systemic inflammation or metabolic syndrome, please.

**The study does not consider the impact of antimalarial or antidiabetic medications, like artemisinin derivatives or metformin, which can influence glucose metabolism and galectin-3 levels.**

Our study was a cross-sectional study, which lack the capacity to examine this research question, please. We focused on active malaria without any prior treatment, please. In this case, impact of antimalaria was never a focus, please. In terms of the diabetics and metformin, it was never part of our plan, as we believe such a question can be properly answered in a longitudinal study, please. Therefore, we are of the respectful view that these issues are outside the scope of the current manuscript, please.

**21. Insulin and glucose levels can vary throughout the day. Were samples collected during a standardized fasting window? If not, variability in sample timing could have affected HOMA-IR estimates.**

Yes, samples were taken during the standardized biochemical fasting window, please.

**Reviewer #2: The current study is relevant since diabetes is on the rise in the African region where malaria is highly endemic. Here the authors have made an attempt to find the role of galectin-3 with diabetes and malaria. They did not find concrete evidence the exact role galectin-3 irrespective of diabetes status, malaria and insulin resistance. Some suggestions:
1. Please mention whether the lifestyle is more responsible for diabetes and more so in the elderly participants.**

Please, the data in our current study do not support any information on the cause of diabetes. This is due to the fact that we dealt with individuals who had already developed type 2 diabetes mellitus, please. Thus, our study did not focus on the cause of diabetes. However, literature evidence abound to show that lifestyle factors may be more responsible for diabetes mellitus, especially, in the elderly, please.

**2. Please mention the exact level of parasitaemia and species of malaria.**

Please, this has been done at the discussion section (page 18, paragraph 1).

**References**

# Adatsi R, Pappoe F, Bockarie AS, Derkyi-Kwarteng L, Nsiah P, et al. Falciparum malaria is associated with risk markers of type 2 diabetes mellitus in individuals with or without   COVID-19 exposure. AJEM,2024;25(1):6-16.

#### Adatsi RK, Bockarie AS, Derkyi-Kwarteng L, Pappoe F, Nsiah P, et al. Increased insulin resistance with reduced beta cell function in recovered COVID-19 Ghanaians. Endocr Metab Sci. 2023; 13, 100150. <https://doi.org/10.1016/j.endmts.2023.100150>.

Bahreiny, S.S., Ahangarpour, A., Aghaei, M. et al. A closer look at Galectin-3: its association with gestational diabetes mellitus revealed by systematic review and meta-analysis. J Diabetes Metab Disord. 2024;23,:1621–33.

Bohn-Gettler, CM, & Rapp DN. Depending on My Mood: Mood-Driven Influences on Text Comprehension. J Educ Psychol.2011;103(3):562–77.

Chen M, Zhu B, Chen D, Hu X, Xu X, Shen W-J, et al. COVID-19 may increase the risk of insulin resistance in adult patients without diabetes: a 6-month prospective study. Endocr Pract 2021; 27(8): 834-41.

Foko LPK, Narang G, Tamang S, Hawadak J, Jakhan J, et al. The spectrum of clinical biomarkers in severe malaria and new avenues for exploration. Virulence. 2022;13(1):634-53.

Hamedi SM, Pishghadam R, Fadardi JS. The contribution of reading emotions to reading comprehension: the mediating effect of reading engagement using a structural equation modeling approach. Educ Res Policy Pract. 2020;19:211–38.

He J, Li X, Luo H, Li T, Zhao L, Qi Q, et al. Galectin-3 mediates the pulmonary arterial hypertension-induced right ventricular remodeling through interacting with NADPH oxidase 4. J Am Soc Hypertens. 2017;11(5):275-89.e2.

Li P, Liu S, Lu M, Bandyopadhyay G, Oh D, et al. Hematopoietic-derived galectin-3 causes cellular and systemic insulin resistance. Cell. 2016;167(4):973-84.e12.

Li Y, Li T, Zhou Z, Xiao Y. Emerging roles of galectin-3 in diabetes and diabetes complications: a snapshot. Rev Endocr Metab Disord. 2022;23(3):569–77.

Matthews N, Folivi F. Omit needless words: Sentence length perception. PLoS One. 2023;18(2):e0282146.doi:10.1371/journal.pone.0282146

Oakley MS, Majam V, Mahajan B, Gerald N, Anantharaman V, et al. Pathogenic roles of CD14, galectin-3, and OX40 during experimental cerebral malaria in mice. Plos One. 2009;4(8):e6793. <https://doi.org/10.1371/journal.pone.0006793>.

#### Obeng JA, Adatsi RK, Derkyi-Kwarteng L, Bockarie AS, Nuvor SV, et al. Increased diabetogenic risk of recovered COVID-19 infection and unexposed respondents in the Central Region of Ghana. Sci Afri. 2024;24:e02179. <https://doi.org/10.1016/j.sciaf.2024.e02179>.

Ohkura T, Fujioka Y, Nakanishi R, Shiochi H, Sumi K, et al. Low serum galectin-3 concentrations are associated with insulin resistance in patients with type 2 diabetes mellitus. Diabetol Metab Syndr. 2014;6(1):1–7.

Scrimin, S., & Mason, L. Does mood influence text processing and comprehension? Evidence from an eye-movement study. Br J Educ Psychol. 2015;85:387–406.
