# Supplementary material for: Galectin-3 did not associate with malaria-related insulin resistance in diabetic and non-diabetic respondents at a Ghanaian General Hospital
Source: PLoS One. 2025 Aug 13;20(8):e0330068. doi: 10.1371/journal.pone.0330068 (PMC12349726; doi:10.1371/journal.pone.0330068)
Supplement: Attachment — Submitted filename: RESPONSE TO REVIEWERS COMMENTS.docx [file pone.0330068.s003.docx]

**RESPONSE TO REVIEWERS COMMENTS**

Please, the specific comments of our esteemed Reviewers are presented in bold Arial font size 12 and aligned with the left margin. However, our responses to these specific comments are in font size 12 of Times New Roman, unbolded and justified, please, for ease of reference and clarity.

**Thank you very much for your thorough and comprehensive responses to my previous comments and suggestions. I appreciate the effort you've put into addressing the feedback.
I also took note of your comments regarding sentence length, my emotions and mood, and the background of English learners, and I have considered them while reviewing the revised version.
As mentioned earlier, I recommend seeking assistance from a native English speaker to help improve the readability and clarity of your manuscript.**

We have noted the maintenance of this view by our esteemed Reviewer and have had a Professor of English Language to peruse and pass our revised manuscript, please. We believe in recognized expertise in the English language instead of just being a native speaker, please. Moreover, our institution is endowed with a number of scholars in the English language. As we indicated in our earlier response, a colleague with expertise in the English language did not find anything challenging with our write-up. A native English speaker without any formal advanced training in the English language, in our view, is inappropriate to examine a scientific manuscript of this nature. Therefore, a recognized scholar of the highest repute in an academic institution like ours should be the most appropriate person to examine the manuscript, please. It is our hope that with this information, our esteemed Reviewer will be comfortable in agreeing with us on the English language suitability of our manuscript, please. Above all, the acknowledgement by our esteemed Reviewer of the comprehensive nature of our earlier responses to the previous comments connotes our ability to communicate effectively to our audience.

In spite of the above, we have appropriately employed the services of our colleague with advanced expertise in the English language to peruse our revised manuscript in line with the suggestion by our esteemed Reviewer, please. With this, we believe there should be no issue with language suitability of our revised manuscript, please.

**Below are additional comments that reflect a more detailed and precise reading of the manuscript:**

**1. The term “chimeric” is used without any accompanying explanation of galectin-3’s structural characteristics. If this term is retained, please include a brief description of its chimeric structure; otherwise, consider omitting it to avoid confusion.**

We have retained the term chimeric and elaborated on the structural aspect of galectin-3 as suggested, please (page 3 of the revised manuscript).

**2. Please edit and make improvements. Figure 1: The current version is asymmetrical and might not be up to the target journal's visual standards.**

We have edited the figure 1 to make it acceptable to the journal using the Preflight Analysis and Conversion Engine (PACE) digital diagnostic tool in line with the journal’s requirements, please.

**3. The sentence “In addition, the region has the lowest prevalence of diabetes at 4.5%, but the highest proportion of undiagnosed cases at 53.6%” lacks a supporting reference. Please add a citation.**

Please, this has been done in the revised manuscript (page 3).

**4. Causality and Study Design: While the study is cross-sectional, some phrases (e.g., “insulin resistance could be predicted by glucose and insulin”) imply causality. Such interpretations should be avoided in non-interventional studies. Use cautious language like “associated with.”**

We used the word ‘predicted’ in the context of logistic regression. However, we have modified it as suggested in the revised manuscript, please (pages 2, 18 & 19).

**5. The statement “Galectin-3 was postulated to promote cerebral malaria…” refers to experimental work but does not distinguish between findings from animal models and human studies in Ghana. Please provide appropriate context and caveats.**

Please, this sentence has been modified appropriately for the needed context and caveats, in the revised manuscript (page 3).

**6. Correct the spelling of “glectin-3” to “galectin-3” at P4.**

Please, this has been done (page 4 of the revised manuscript).

**7. Phrases like “averting severe illness” or “curbing the menace” are overly informal. Please consider more scientific alternatives, such as “mitigating disease severity” or “addressing disease burden.”**

Please, we have replaced ‘averting’ with “preventing” and ‘curbing’ with “reducing” (page 4 of the revised manuscript). With these changes, we believe that the expected formal scientific presentation has been met, please.

**8. The paragraph introducing galectin-3 rapidly shifts between multiple diseases (T2DM, cancer, asthma, etc.). For coherence and relevance, consider narrowing the focus to metabolic and infectious diseases.**

We have carefully considered this suggestion by our esteemed Reviewer, and appreciate the call to narrow the focus to metabolic and infectious diseases. However, we are of the respectful view that precision is needed here because not all infectious or metabolic diseases have been associated with galectin-3 per the available literature, please. Therefore, there is the need to indicate the exact conditions as was done in the earlier versions and maintained in the revised version of the manuscript, please. In addition, asthma (Serafino-Agrusa et al., 2015; Pite et al., 2020) and cancer (Coller, 2014; Seyfried et al., 2014; Wishart, 2015; Gyamfi et al., 2022) are associated with some components of metabolic syndrome but there is no consensus on their classification as metabolic diseases in spite of emerging evidence to their metabolic nature. We believe that specifying the exact conditions portrays the versatile nature of galectin-3 in associating with various diseases, please.

**9. The introduction could be strengthened by acknowledging limitations or controversies in the existing literature—such as variability in galectin-3 levels due to comorbidities or unclear causative relationships in prior studies.**

We have carefully considered this view by our esteemed Reviewer with regards to this suggestion, please. However, we are of the respectful view that such a bold posture should emanate from a comprehensive review of the existing literature on galectin-3 and its relationship with diabetes and related comorbidities. Per our limited review of the relevant literature on the relationship of galaectin-3 with malaria and diabetes mellitus, we do not believe that we are clothed with enough information to make such a declaration, please. We prefer things to be maintained as reflected in the revised manuscript, please.

**10. While the use of simple random sampling is appropriate, it should be acknowledged that hospital-based recruitment may introduce selection bias.**

This acknowledgement has been made in the revised manuscript, please (page 23).

**11. Please clarify whether malaria parasitemia (e.g., parasite density) was quantified. Severity stratification would add significant value beyond a binary malaria diagnosis.**

Parasitaemia was determined microscopically, quantified and reported as such in the previous version of the manuscript and maintained in the revised version (pages 8, 9, 12 & 13), please. However, we did not stratify severity of the signs and symptoms, please. By the design of our study, which focused on the disease. We did not collect any data on disease severity, please.

**12. Reiterate clearly that causality cannot be inferred due to the cross-sectional design.**

This was clearly acknowledged as a limitation in the earlier version and has been maintained on page 23 of the revised manuscript, please. However, the word difficult has been replaced with impossible in the revised version, please.

**13. While sample size calculation is addressed, please ensure that effect sizes are also reported in the results.**

This has been addressed in the revised manuscript (pages 2 & 9-15), please. Published (Serdar et al., 2021) thresholds were used in interpreting the effects sizes with adoption of the standard interpretations, please.

**14. The phrase “Seven millimetres of venous blood” should be corrected to “Seven millilitres.”**

This error has been corrected as suggested, please (page 6 of revised manuscript).

**15. The use of TMB and dual-wavelength readings (450 and 570 nm) is common, but a brief rationale should be included to enhance clarity for reproducibility.**

Please, the suggested clarity has been provided (pages 7 & 8 of the revised manuscript).

**16 Consider shortening overly technical assay descriptions. Emphasize essential elements such as the kit name, detection principle, sensitivity, and standard curve methodology.**

This suggestion by our esteemed Reviewer is laudable. However, for the sake of the principle of enhanced clarity, coupled with the absence of any breach of the Journal’s policy regarding length or number of words of the manuscript, we kindly implore that the current details of the assay descriptions be maintained, please.

**17. While the limitations of a cross-sectional design are mentioned, causal phrases such as galectin-3 “worsening” hyperglycemia should be revised to more appropriate wording like “associated with.”**

This suggestion has been duly incorporated in the revised manuscript (page 21), please.

**18. The explanation suggesting that lower parasite load in diabetics reflects immune dysfunction is speculative. This should be expanded or supported with relevant data (e.g., immune profiling or prior malaria exposure).**

We have carefully considered this observation by our esteemed Reviewer and we respectfully disagree with the view that our explanation is speculative, please. That explanation, in our view, has a sound scientific basis. In the current study, we focused on disease not just infection. Diabetics who had malaria showed lower parasite levels (3819/µl vs 9981/µl; P < 0.001; table 2) compared with the parasite levels of non-diabetics who had malaria (table 2, pages 12-15 of revised manuscript). However, the two groups did not differ in the levels of total white blood cells or any of the components of the white blood cell (P > 0.05; table 2). Immunity to malaria as a disease is conferred by the overall effect of the function of the white blood cells. Development of signs and symptoms for malaria following an infective bite by a mosquito depends on the overall response of the immune system to the *Plasmodium* parasite. If one’s immune system is strong enough, it should take more parasites to develop the needed signs and symptoms with time. However, if one’s immune system is not that strong, even few parasites are enough to induce the needed signs and symptoms of the disease. The reduced parasite level in diabetics compared with non-diabetics has been observed by several studies (Ikekhazu et al., 2010; Ndiok et al., 2016) and has been ascribed to cytotoxic effects of glucose-induced peroxides (Chattopadhyay et al., 2015; Msolly et al., 2016; Tian et al., 2017; Ghoshal et al., 2016) which is heightened in the diabetic state. This peroxide-based cytotoxicity is an acknowledged mechanism of action of some antimalarials (Siddiqui et al., 222).

In persons with strong immune system and cytotoxic effects on parasites to result in lower level of parasites, clinical signs and symptoms should not develop. However, because the overall immune system of diabetics is relatively weak (Berbudi et al., 2020; Alexander et al., 2024), the relatively low parasite level was still able to induce the needed clinical signs and symptoms of malaria. Even studies that that have reported higher parasite levels and malaria severity in diabetes patients (Danquah et al., 2010; Thatoi, 2018) compared to controls reinforce the concept of impaired immunity in the diabetic group. This is because the higher parasite level could be ascribed to enhanced parasite growth due to the high glucose level in diabetics because the *Plasmodium* parasite lacks the capacity for glucose synthesis (Srivastava et al., 2016). As a result, it relies on exogenous glucose, which is readily available in the diabetic host. In addition, the impaired nature of the immune system of diabetics may impair parasite clearance and allow for increased parasite levels. Thus, whichever direction is considered, the view of weakened immunity is plausible. In our case, we considered the malarial disease without stratification to severity and our finding in our view supports the concept of weakened immunity in diabetics (Danquah et al., 2010; Thatoi, 2018; Carrillo-Larco et al., 2019).

In spite of the above justification of our view of weakened immunity in diabetes, we have expanded the discussion (page 22 of revised manuscript), in line with the suggestion by our esteemed Reviewer, please.

19. Avoid repetitive phrases like “in our context” or “in the current study” unless necessary for clarity.

We have carefully noted this suggestion and have avoided it in unnecessary contexts, please. Therefore, those left in the revised manuscripts are very necessary for the appropriate comprehension of the manuscript, please.

**Reviewer #2: The MS is OK now. The authors have addressed all the comments raised by the reviewers. Only I suggest to mention that falciparum is responsible for hypoglycemic conditions. Here how you will explain this in view of the present study.**

We have carefully considered this laudable suggestion by our esteemed Reviewer and are of the respectful view that the mention of *falciparum* being responsible for hypoglycaemic conditions is inapplicable in our context, please. Although we acknowledge the role of *falciparum* malaria in hypoglycaemic conditions, not all *falciparum* malarial conditions result in hypoglycaemia. Mild and moderate *falciparum* malarial conditions such as observed in the current study do not give rise to hypoglycaemia. It is rather the severe (Shalev et al., 1992; Zijlmans et al., 2008; Ogetii et al., 2010) and cerebral (Atabani et al., 1990; Bondi, 1992; Idroet al., 2006) forms of malaria that normally result in hypoglycaemia. In diabetics for example, hypoglycaemia can occur as a result of inappropriate medication dosage even without malaria. Pregnancy, which is a normal physiological phenomenon may be associated with hypoglycaemia. In the current study, the high level of parasitaemia that is normally associated with hypoglycaemia was not found in any of our participants. Our participants with diabetes for instance demonstrated a lower parasite load but a higher glucose level than controls. If we had per adventure observed a hypoglycaemic situation in any of our participants, especially, those with diabetes mellitus, we would have explained it to be due to a possible side effects of a given medication or erroneous dosage coupled with high parasite load, please. Thus, the nature of malaria patients involved in this study is such that hypoglycaemia is not expected, please.

**REFERENCE**

Alexander M, Cho E, Gliozheni E, Salem Y, Cheung J, Ichii H. Pathology of diabetes-induced immune dysfunction. International J Mol Sci. 2024; 25(13):7105. <https://doi.org/10.3390/ijms25137105>.

Atabani GS, Saeed BO, elSeed BA, Bayoumi MA, Hadi NH, Abu-Zeid YA, Bayoumi RA. Hypoglycaemia in Sudanese children with cerebral malaria. Postgrad Med J. 1990; 66(774):326-7.

Berbudi A, Rahmadika N, Tjahjadi AI, Ruslami R. Type 2 diabetes and its impact on the immune system. Curr Diabetes Rev. 2020;16(5):442-9.

Bondi FS. The incidence and outcome of neurological abnormalities in childhood cerebral malaria: a long-term follow-up of 62 survivors. Trans R Soc Trop Med Hyg. 1992; 86(1):17-9.

Carrillo-Larco RM, Altez-Fernandez C, Ugarte-Gil C. Is diabetes associated with malaria and malaria severity? A systematic review of observational studies. Wellcome Open Res. 2019;4(136). <https://doi.org/10.12688/wellcomeopenres.15467.3>

Chattopadhyay M, Khemka VK, Chatterjee G. Enhanced ROS production and oxidative damage in subcutaneous white adipose tissue mitochondria in obese and type 2 diabetes subjects. Mol Cell Biochem. 2015;399: 95-103.

Coller HA. Is cancer a metabolic disease?.  Am J Pathol.2014;184(1):4–17.

Danquah I, Bedu-Addo G, Mockenhaupt FP. Type 2 diabetes mellitus and increased risk for malaria infection. Emerg Infect Dis. 2010;16(10):1601-4.

Ghoshal K, Das S, Aich K, Goswami S, Chowdhury S, Bhattacharyya M. A novel sensor to estimate the prevalence of hypochlorous (HOCl) toxicity in individuals with type 2 diabetes and dyslipidemia. Clinica Chimica Acta; Int J Clin Chem. 2016; 458:144–153. https://doi.org/10.1016/j.cca.2016.05.006

Gyamfi J, Kim J, Choi J. Cancer as a metabolic disorder. Int J Mol Sci. 2022;23(3):1155. https://doi.org/10.3390/ijms23031155

Idro R, Carter JA, Fegan G, Neville BG, Newton CR. Risk factors for persisting neurological and cognitive impairments following cerebral malaria. Arch Dis Child. 2006;91(2):142-8.

Ikekhazu EJ, Neboh EE, Nwobodo MW. Type-2 diabetes mellitus and malaria parasitemia: Effect on liver function tests. Asian J Med Sc, 2010 214-7.

Msolly A, Miled A, Kassab A. Hydrogen peroxide: an oxidant stress indicator in type 2 diabetes mellitus. J Cardiovasc Dis. 2013;1(2): 48-52.

Ndiok E O, Ohimain E I, Izah SC. Incidence of malaria in type 2 diabetic patients and the effect on the liver. J Mosq Res. 2016;6(15):1-8.

Ogetii GN, Akech S, Jemutai J, Boga M, Kivaya E, Fegan G, Maitland K. Hypoglycaemia in severe malaria, clinical associations and relationship to quinine dosage. BMC Infect Dis. 2010; 10:334. https://doi.org/10.1186/1471-2334-10-334

Pite H, Aguiar L, Morello J, Monteiro EC, Alves AC, Bourbon M, Morais-Almeida M. Metabolic dysfunction and asthma:Ccurrent perspectives. J Asthma Allergy. 2020;13:237–47.

Serafino-Agrusa L, Spatafora M, Scichilone N. Asthma and metabolic syndrome: Current knowledge and future perspectives. World J Clin Cases. 2015; 3(3):285–92.

Serdar CC, Cihan M, Yücel D, Serdar MA. Sample size, power and effect size revisited: simplified and practical approaches in pre-clinical, clinical and laboratory studies. Biochem Med (Zagreb) 2021;31(1):010502. https://doi.org/10.11613/BM.2021.010502

Seyfried TN, Flores RE, Poff AM, D'Agostino DP. Cancer as a metabolic disease: implications for novel therapeutics. Carcinogenesis.2014;35(3):515–27.

Shalev O, Tsur A, Rahav G. Falciparum malaria-induced hypoglycaemia in a diabetic patient. Postgrad Med J.1992; 68: 281-2.

Siddiqui G, Giannangelo C, De Paoli A, Schuh AK, Heimsch KC, Anderson D, et al. Peroxide antimalarial drugs target redox homeostasis in *plasmodium falciparum* infected red blood cells. ACS Infect Dis. 2022;8(1), 210–26.

Srivastava A, Philip N, Hughes KR, Georgiou K, MacRae JI, Barrett MP, et al. Stage-specific changes in plasmodium metabolism required for differentiation and adaptation to different host and vector environments. PLoS Pathog.2016;12(12): e1006094. <https://doi.org/10.1371/journal.ppat.1006094>

Thatoi PK. Diabetes and severe malaria—clinical profile and outcome. Diabetes. 2018;67 (Supplement_1): 2388–PUB. https://doi.org/10.2337/db18-2388-PUB.

Tian R, Ding Y, Peng YY, Lu N. Myeloperoxidase amplified high glucose-induced endothelial dysfunction in vasculature: Role of NADPH oxidase and hypochlorous acid. Biochem Biophys Res Commun. 2017;484(3):572-8.

Wishart, D. S. (2015). Is Cancer a Genetic Disease or a Metabolic Disease? eBioMedicine, *2*(6), 478 – 479.

Zijlmans W, van Kempen A, Ackermans M, de Metz J, Kager P, Sauerwein H. Glucose kinetics during fasting in young children with severe and non severe malaria in Suriname. Am J Trop Med Hyg. 2008, 79(4):605-12.
